# The Study of Muscle, Mobility, and Aging (SOMMA): An Overview

**DOI:** 10.1093/geroni/igab046.480

**Published:** 2021-12-17

**Authors:** Steve Cummings, Peggy Cawthon, Russell Hepple

## Abstract

SOMMA is an NIA-funded cohort study to identify biological determinants of mobility and fitness. The overall aim of SOMMA is to use biopsies, novel biomarkers, advanced imaging, and intensive physical and cognitive assessments to elucidate the biological processes that contribute to changes in mobility and physical fitness with aging. SOMMA will recruit 875 people age 70+ (of whom about 200 have been enrolled.) We take biopsies of the vastus lateralis muscle to quantify mitochondrial content and function of the electron transport chain. We use 31PMR spectroscopy to quantify mitochondrial capacity to generate ATP in quadriceps muscle (ATPmax). We will quantify other biological properties in biopsies including denervation, autophagy and accumulated biochemical damage and use gene expression to discover pathways that contribute to mobility and fitness. SOMMA uses MR for quadriceps volume and D3Cr dilution for total skeletal muscle mass, cardiopulmonary exercise testing to measure fitness (VO2 peak). We are also making many other intensive assessments of physical and cognitive function. Mobility endpoints include baseline and three year change in 400 m and 4 meter gait speed and fitness. SOMMA is building a large biobank of muscle, adipose blood, and urine specimens that will be available for ancillary studies. In this Symposium, we will present results from analyses of associations between muscle mitochondrial function and strength, muscle mass, cognitive performance, gait speed, and fitness. The symposium will also preview opportunities for collaborations and ancillary studies with SOMMA.

